# Negatively Charged Submicron Heterogeneities in Aqueous Solutions of Biomolecules as Alkaline Membraneless Organelles

**DOI:** 10.3390/ijms27136015

**Published:** 2026-07-04

**Authors:** Nadezda Penkova, Natalia N. Rodionova, Nikita V. Penkov

**Affiliations:** 1 Institute of Cell Biophysics RAS, Federal Research Center Pushchino Scientific Center for Biological Research of the Russian Academy of Sciences, Moscow Region, Pushchino 142290, Russia; kokanchik@rambler.ru; 2 OOO “NPF “MATERIA MEDICA HOLDING”, Moscow 129272, Russia; rodionovann@materiamedica.ru

**Keywords:** submicron heterogeneities, membraneless organelles, biomolecular interactions, structure of water solution, particle charge, isoelectric point

## Abstract

In this work, charge characteristics of submicron heterogeneities (SMH) spontaneously formed in aqueous solutions of various biomolecules: seven amino acids of various types (nonpolar glycine, polar serine, hydrophobic valine, aromatic phenylalanine, sulfur-containing methionine, glutamic acid and basic arginine), ATP, monosaccharide glucose and disaccharide sucrose were studied. The isoelectric points of the SMH in the amino acid solutions determined turned out to be in the pH range from 2.4 to 4, being shifted to the acidic region relative to the isoelectric points of the amino acids themselves (except for glutamic acid). The zeta potential of the SMH was measured in solutions of all the biomolecules under conditions close to the intracellular environment at pH = 7 and basic K^+^ ion content 150 mM. The zeta potential appeared to be negative in all cases. Using these values of the zeta potential, the concentration of OH-anions inside the SMH was estimated, and the pH values corresponding to this concentration turned out to be in the range of 7–10. Since the cell cytosol is an aqueous solution of various biomolecules, SMH must also form inside cells. An analogy is drawn between SMH and membraneless organelles, many of which have been discovered recently. The presence of compact regions with alkaline pH inside the cell is a fundamentally new factor in cell biology, which may have important consequences.

## 1. Introduction

In chemistry, the phenomenon of stratification of solutions is well known, when several stable phases form in a homogeneous solution under certain conditions. In chemical thermodynamics [1], this is described by phase diagrams. Many examples are known, for instance, solid precipitation in supersaturated salt solutions, separation of a homogeneous aqueous solution of phenol into two liquid phases [2], micelle formation in aqueous solutions [3], and many others. In these cases, the coexisting phases have significant differences in the chemical composition and physical properties. At the same time, there are many homogeneous solutions called true solutions.

An interesting observation was made in 1972 with one of these solutions, water-tretbutanol, which is still considered as a true solution [4]. Based on the features of light scattering, the presence of certain heterogeneities was established in it. Despite the initial criticism [5], this was later confirmed many times [6,7,8]. In addition, heterogeneities were also identified in hundreds of other solutions considered to be true [9,10,11]. In the vast majority of cases, heterogeneities were observed in aqueous solutions of low-molecular-weight organics, which is apparently a universal characteristic of such solutions. Thus, many heterogeneous solutions are considered true, which looks like a contradiction by definition. This is explained by the fact that, firstly, these heterogeneities are difficult to detect, and secondly, their nature is poorly understood. Therefore, in many cases it is easier to ignore them.

Many methods used in colloidal chemistry, for example osmometry [9], small-angle X-ray scattering [12], and small-angle neutron scattering [13], are not sensitive to such heterogeneities. They can only be reliably detected using dynamic light scattering (DLS) and multi-angle static light scattering [9,10,14,15,16]. Using a number of examples, we have shown that the refractive index of these heterogeneities differs from the medium by ~0.001–0.01, and the density by ~1% [17,18], and their sizes are hundreds of nanometers. This explains the inconspicuousness of these heterogeneities.

Taking into account the indicated submicron size, which is typical for such heterogeneities in a wide variety of solutions, in our works [17,19,20] they were called submicron heterogeneities (SMH). They have also been called by other names: supramolecular structures or domains [9], large-scale heterogeneities [21], mesoscopic droplets [22], or solvophobicity-driven mesoscale structures [23].

SMH is known to be stable for many months [10,23]. Several explanations have been proposed for the formation and stability of SMH: the bonding of organic molecules through hydrogen-bonded bridges from water molecules [11], spinodal decomposition of solution under the influence of dichotomous noise of twinkling hydrogen bonds [16], or the formation of SMH with the participation of hydrophobic components [12,13,15]. The latter option implies the presence of a trace amount of hydrophobes [7,23,24], which is present in almost any solution, due to the presence of dissolved air molecules, the use of plastic labware and impurities in reagents.

In some studies, SMH were interpreted as air bubbles [25,26,27,28], which was apparently done by analogy with another well-known phenomenon of the formation of babstones in aqueous-salt solutions [29]. We have shown that submicron air bubbles can indeed be present in organic solutions, but they make up only a small fraction of the observed submicron fraction [17].

In a two-component mixture, SMH can occur only as a result of an inhomogeneous distribution of the components. This means that in aqueous solutions of organic matter, SMH must be formations with an increased concentration of organic matter. According to the estimates of our work, in solutions of sugars, amino acids and nucleotides with a concentration of about 100 mM, the concentrations of these molecules in SMH are several times higher [17,18]. That is, SMH are some kind of clumps of organic molecules; however, they cannot be correlated with any type of aggregates in which the molecules are directly connected to each other [9]. Rather, they are “clouds” with an increased concentration of organic molecules.

Despite the limited knowledge of SMH, understanding their presence in solutions opens up great prospects to the possible change in some paradigms. Thus, taking into account the SMH, new ways of describing chemical reactions were proposed [30,31,32,33,34], some effects of microfluidics were explained [19], and ideas of some applications were even suggested [35]. It is especially interesting to consider the importance of SMH in biological processes. In our work, it was shown that SMH are formed in aqueous solutions of all low-molecular-weight biomolecules, including nucleotides [20], amino acids [36], and sugars [17,18]. The fourth and final type of biomolecule, phospholipids, can be included in this list. They also form submicron formations—liposomes, the basis of biological membranes. However, liposomes have been well studied and are formations of a different type, with direct binding of phospholipid molecules [3,37]. Thus, all types of low-molecular biomolecules in water are capable of organizing into submicron formations, although this has not yet become a generally accepted fact.

It is possible to move on to larger biomolecules. When analyzing sizes of globular proteins in solutions using the DLS method, in addition to the fraction of protein molecules, an additional fraction of hundreds of nm is almost always detected in the nanometer scale. Experts explain this in the standard way: by the presence of impurities, protein aggregates or air bubbles [38,39,40]. But the fact that any protein solution, regardless of purity, contains a certain submicron fraction clearly indicates a certain universal physico-chemical phenomenon.

At the same time, in recent years, a lot of data on membraneless organelles have appeared in cell biology: P bodies [41], nuclear speckle [42], PML nuclear body [43], nuclear stress body [44], and Paraspeckles [45]. They play a significant biological role. It is generally accepted to explain their occurrence by liquid–liquid phase separation with the formation of regions with sizes of ~0.1–1 µm, enriched in proteins and nucleic acids [46,47,48,49]. But there is no full-fledged explanation for this phenomenon in thermodynamic terms, and there is no full-fledged explanation for the formation of SMH.

As can be seen, the discussed SMH and membraneless organelles have a lot in common, which has not been noticed in the literature before. Their only difference is the contrast. Organelles are experimentally more noticeable. At the same time, SMH are naturally present in the cell, since the cytosol contains a large number of different biomolecules that form SMH in aqueous solutions. If all types of SMH are identified, it is likely that there will be many more membraneless organelles than are currently known.

For any colloid, in addition to size and chemical composition, another important characteristic is the charge. We were not aware of any work devoted to the determination of the charge of membraneless organelles. At the same time, the charge of SMH in the solutions of some biomolecules was analyzed and in all cases they turned out to be negative [19,36,50]. To date, the mechanism of acquiring the charge of SMH, its dependence on the composition of the solution, its contribution to colloidal stability, and, of course, its possible biological significance, are unclear.

In this paper, the charge characteristics of SMH in solutions of a number of low-molecular-weight biomolecules are analyzed: seven amino acids, ATP, and two sugars. In the aqueous solutions under consideration, the charge is mainly formed due to proton transfer, which should lead to a local change in pH. The implications of charged SMH for cell biology are discussed.

## 2. Results and Discussion

**Figure 1:**
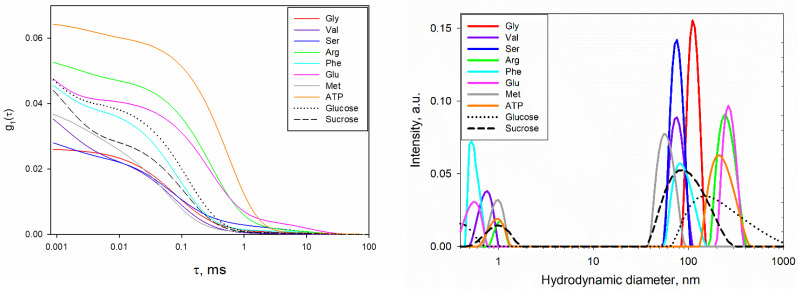
Correlation functions (on the **left**) and size distributions (on the **right**) of optical heterogeneities in aqueous solutions of seven amino acids, ATP, and two sugars. (The correlation function of the ATP solution is reduced by 10 times for the convenience of comparative analysis.)

Figure 1 shows correlation functions and size distributions of optical heterogeneities in the studied biomolecule solutions. In addition to the molecular fraction of about 1 nm [12,14], the SMH fraction with a hydrodynamic diameter of about 100–300 nm is observed in all solutions. The distributions have some variation, but this paper did not aim to analyze the sizes of the SMH, since the exact sizes of the SMH (hydrodynamic and geometric) were determined in our other works [17,18]. The distributions in Figure 1 are shown only to confirm the presence of SMH in all analyzed solutions.

It has been shown that SMH in some solutions of biomolecules have a negative ζ-potential, that is, they are negatively charged [19,20,36]. After filtration of these solutions, the ζ-potential could not be determined, which means that the measured zeta potential referred specifically to the SMH, and not to individual biomolecules. At the same time, many biomolecules are themselves charged. For example, amino acids in water have a pH-dependent charge, which is 0 only at the isoelectric point (Table 1). According to the general laws of colloidal chemistry, SMH should also change their charge when the pH changes, and at a certain pH their charge will reset to zero. Colloidal particles have a complexly organized double electric layer, and their isoelectric point is determined by zeroing the ζ-potential [51]. For all the amino acid solutions studied, the isoelectric points of the SMH were determined, which are shown in Table 1.

The data obtained indicate that the isoelectric points of the SMH in amino acid solutions are located at lower pH values than the isoelectric points of the amino acids themselves. The only amino acid for which this could not be reliably shown is glutamic acid.

For all the amino acids considered, the isoelectric points of the SMH are in the acidic range of pH 2.4–4. Note that the selected amino acids cover all the main chemical types: nonpolar glycine, polar serine, hydrophobic valine, aromatic phenylalanine, sulfur-containing methionine, glutamic acid and basic arginine. Apparently, the presence of SMH pI in the acidic region is typical for all amino acids, including basic (arginine) and acidic (glutamic acid) amino acids. This indicates the common nature of the formed SMH in aqueous solutions of amino acids. Accordingly, at higher pH values, the charge of the SMH is negative. And even in an arginine solution, the molecules of which are positively charged up to pH = 10.8, SMH have a negative charge at pH > 4.

It is most interesting to consider the charge characteristics of SMH in solutions of biomolecules close to the intracellular environment. For this purpose, the ζ-potential of SMH was measured in solutions of seven amino acids, ATP and two sugars at pH = 7 in water and with the addition of 150 mM KCl (Table 2).

As can be seen, the ζ-potentials of SMH in solutions of all biomolecule types are negative at a neutral pH. This indicates the commonality of the charge separation process between the phase of a true solution of various biomolecules and the SMH formed in them. As the ionic strength of the solutions increases, the ζ-potential decreases slightly (Table 2), which is explained by the well-known phenomenon of ion charge shielding. Nevertheless, under conditions close to the intracellular environment in pH (7) and the main ionic composition (150 mM K^+^), the charge of the SMH remains negative for all types of biomolecules. This can only be due to the increased concentration of OH-anions. This means that an alkaline medium is formed inside the SMH. We will evaluate the pH in the SMH by calculating the content of OH-anions in them from the measured values of the ζ-potential.

ζ-potential is the potential on the slipping plane, which is somewhat removed from the real surface of the SMH. The counterions within the slipping plane are strongly associated with the SMH and partially shield its charge. Therefore, the absolute value of the potential on the surface of the SMH, *φ*_0_, is slightly greater than the ζ-potential. SMH have a spherical shape [17,18], so the Overbeek formula [53] is applicable:

(1) $$\varphi =\frac{4RT\gamma }{zF}e^{-x/d},$$

*φ* is the potential depending on the distance *x* to the surface of the SMH, *R* is the gas constant, *T* is the absolute temperature, *z* is the charge of the counterions in units of elementary charge, *F* is the Faraday constant, *d* is the Debye length, and *γ* is determined as follows:

(2) $$\gamma =\left(exp\left(\frac{zF\varphi_{0}}{2RT}\right)-1\right)/\left(exp\left(\frac{zF\varphi_{0}}{2RT}\right)+1\right).$$

The slipping surface corresponding to the ζ-potential is usually removed from the actual surface of the charged particle by a distance of d or slightly less. We can estimate *φ*_0_, assuming that the ζ-potential is realized at a distance d from the surface of the SMH. Then in Formula (1), $e^{-x/d}$ = $e^{-1}$ and Formulae (1) and (2) allow us to associate *φ*_0_ with ζ-potential:

(3) $$\frac{4RT}{zFe}\left(exp\left(\frac{zF\varphi_{0}}{2RT}\right)-1\right)/\left(exp\left(\frac{zF\varphi_{0}}{2RT}\right)+1\right)=\zeta .$$

Equation (3) is solved analytically:

(4) $$\varphi_{0}=\frac{2RT}{zF}ln\left\{\frac{1+\frac{\zeta zFe}{4RT}}{1-\frac{\zeta zFe}{4RT}}\right\}$$

Substituting in Formula (4) the value of *z* = 1, referring to the counterions (K^+^, H^+^, and Na^+^), T (298 °K), and the maximum ζ-potential from Table 2 (−0.025 V), we obtain *φ*_0_ ≈ −0.08 V. If the slipping surface is located at a distance less than d from the SMH surface or |ζ-potential| < 0.025 V, then the value of *φ*_0_ will be slightly less. In this case, the value of 0.08 V is the maximum modulo estimate.

The surface potential of a spherical object, *φ*_0_, is related to its charge, *q*, as follows:

(5) $$\varphi_{0}=\frac{q}{4\pi \varepsilon_{0}\varepsilon r},$$

where $\varepsilon_{0}\approx 8.85\times 10^{-12}\;F/m$ is the electrical constant, *r* ≈ 130 nm is the characteristic geometric radius of SMH [17,18], and ε≈77 is the static permittivity of the solutions with 150 mM KCl [51]. Using Formula (5) and the calculated value of *φ*_0_ ≈ −0.08 V, we calculate *q* ≈ −8.9 × 10^−17^ Q, which corresponds to the number of elementary charges in the form of OH-anions: N ≈ 560. This number of ions (the maximum estimate) is present in a single SMH.

A part of these ions is located near the inner surface of the SMH, forming a double electric layer together with the outer counterions. However, most of these ions are in the volume of SMH due to the fact that the thermal energy $kT=4.14\times 10^{-21}$ J is much higher than the Coulomb repulsion energy of charges, *W_q_*, located at opposite points on the surface of the SMH (at a distance of 2*r* ≈ 260 nm): $W_{q}={\left(1.6\times 10^{-19}\right)}^{2}/4\pi \varepsilon_{0}\varepsilon 2r$ = 1.15 × $10^{-23}$ J.

If we continue to maximize the concentration of OH-anions, assuming their homogeneous distribution in the volume of the SMH, then their numerical concentration is determined as follows: $N/\frac{4}{3}\pi r^{3}\approx 6\times 10^{19}\;l^{-1}\approx 10^{-4}\;M$. Such a concentration of OH ions, taking into account the ionic product of water 10^−14^, corresponds to a concentration of H^+^ ions equal to 10^−10^, that is, pH = 10. This value is somewhat overestimated, but the data obtained indicate that the medium inside the SMH can be quite strongly alkaline.

As has been shown, SMH are formed in aqueous solutions of almost any biomolecule. Consequently, SMH of various compositions should also be formed in the cytosol of cells. Upon careful consideration, SMH may well replenish the list of membraneless organelles that has been expanding in recent years [41,42,43,44,45,46,47,48,49]. At the same time, their alkaline medium may have a biological meaning. As we have shown [17,18], SMH are permeable to the liquid environment surrounding them, and their volume concentration in solution can reach ~1–10 vol% [17,18]. The presence of such formations inside the cell should significantly affect the course of various biochemical reactions, including enzymatic reactions. That is, SMH act as submicron reactors with an alkaline medium.

Interestingly, in cell biology, organelles with an acidic medium, such as lysosomes, have long been known. Their pH is 4.5–5, which is 2–2.5 less than the average in a cell. At the same time, organelles with a strongly alkaline environment have not been described. Probably, SMH are those previously undetected alkaline formations in which a commensurate increase in pH relative to neutral can be realized. Since it is not possible to accurately determine the pH inside the SMH based on the available data, as well as in solutions with many components, various SMH are possible, we will limit ourselves to stating that inside the SMH in solutions of biomolecules, an alkaline medium with a pH of more than 7 but less than 10 is realized. This is a fundamentally new factor in cell biology that needs to be registered and understood.

## 3. Materials and Methods

### 3.1. Preparation of Solutions

Aqueous solutions of the following substances were analyzed: D(+)-glucose anhydrous (#131341, Panreac, Barcelona, Spain), D(+)-sucrose (141621, Panreac, Barcelona, Spain), ATP·2Na·3H_2_O (>99%, Dia-M, Moscow, Russia), glycine (>99% Acros organics, Geel, Belgium), DL-valine (>98%, Reachim, Moscow, Russia), DL-β-phenyl-α-alanine (>98%, Reachim, Moscow, Russia), DL-serine (>99%, Reachim, Moscow, Russia), L-glutamic acid (>98%, Reachim, Moscow, Russia), L-arginine hydrochloride (>98%, Reachim, Moscow, Russia), and DL-methionine (>99%, Reachim, Moscow, Russia). Water was used after daily saturation with air at atmospheric pressure and room temperature.

Solutions of ATP and all amino acids except glycine were prepared at a concentration of 50 mM, glycine solution—at a concentration of 100 mM, sugar solutions—200 mM. MilliQ water (Millipore, Darmstadt, Germany) was used for dissolution. The solutions were prepared in 20 mL plastic tubes (Sarstedt, Sarstedt, Germany) at room temperature, while smoothly turning the tubes over without shaking.

The pH of the solutions was changed using HCl (Sigma-Aldrich, St. Louis, MO, USA) and NaOH (Sigma-Aldrich, St. Louis, MO, USA) additives.

Aqueous solutions of these substances were analyzed by themselves and with the addition of KCl (≥99.5%, Sigma-Aldrich, St. Louis, MO, USA) at a concentration of 150 mM.

### 3.2. Measuring Size Distributions

The size distributions of optical heterogeneities in solutions were measured by dynamic light scattering (DLS) using the Zetasizer nano ZS (Malvern Instruments Ltd., Malvern, UK) at a temperature of 25 °C. A laser with a wavelength of 451 nm and a power of 50 mW was used, and light scattering was measured at an angle of 140°. According to the DLS method, to calculate the size distributions, it is necessary to set the correct values of viscosity and refractive index of solutions. These data were taken from the literature: viscosities of amino acid solutions [54,55,56,57], sugars [58], and ATP [59]; and refractive coefficients of amino acid solutions [60], sugars [58], and ATP [61].

### 3.3. Measurement of the ζ-Potential of SMH

ζ-potential of SMH in solutions was measured using the Zetasizer nano ZS (Malvern Instruments Ltd., Malvern, UK) in DTS 1060 cuvettes (Malvern Instruments Ltd., Malvern, UK) at a temperature of 25 °C. In this technique, the optical heterodyning method measures the velocity of charged particles, *v*, in an electric field supplied to the cell by a pair of electrodes. Using *v*, the ζ-potential is calculated according to the formula [51]:

(6) $$\zeta =\frac{3\eta v}{2\varepsilon \varepsilon_{0}Ef_{H}}$$

where *E* is the electric field strength in the solution calculated from the potential difference applied to the electrodes, the distance between the electrodes and the known static permittivity of the solution *ε*; *ε*_0_ is the electrical constant; *η* is the dynamic viscosity of the solution; and $f_{H}$ is the Henry function, depending on the ionic strength and the size of the SMH [51]. For all solutions in which the ζ-potential was measured, $f_{H}$ is close to 1.5. According to the parameter *ε*, all the solutions considered differ from water by no more than 2% [51,58,62], which can be neglected.

### 3.4. pH Measurement

The pH of the solutions was measured using a pH meter included in the MPT-2 autotitrator (Malvern, UK) at room temperature (23–24 °C).

### 3.5. Determination of the Isoelectric Point of SMH in Amino Acid Solutions

The pH value of the solutions gradually changed and the ζ-potential of the SMH was measured. A pH value was searched for at which the ζ-potential is 0. Since the ζ-potential was determined by the velocity of charged particles in an electric field (6), at zero ζ-potential, v = 0 and ζ-potential could not be measured. Considering this, as the ζ-potential approaches zero, the accuracy of its measurement decreases. In this work, the ζ-potential was considered equal to 0 when the measured value fell into the range from −2 to +2 mV. Six repetitions of measurements of each of the 3 newly prepared solutions were performed.

## 4. Conclusions

The charge characteristics of SMH, which spontaneously form in aqueous solutions of a number of low-molecular-weight biomolecules: seven amino acids, ATP, and two sugars, were analyzed. It was found that the pI of SMH in all amino acid solutions, except for glutamic acid, is less than the pI of the amino acids themselves. Under conditions close to the intracellular medium in terms of pH (7) and K+ cations (150 mM), the charge of SMH in solutions of all the biomolecules studied is negative. This indicates a universal and as yet unexplored process of charge separation in solutions of various biomolecules between a molecular solution and SMH. The negative charge of SMH can only be provided by the presence of OH-anions. The concentration of these anions inside the SMH was estimated, which corresponds to an alkaline pH value in the range of 7–10. Since the intracellular medium contains a large number of biomolecules, including those considered in this work, SMH should be formed in it. This makes it possible to put the discussed SMH on a par with many already established membraneless organelles. The presence of stable submicron-sized regions with an alkaline medium should be considered as a fundamentally new factor in cell biology, which has yet to be registered and understood.

## Figures and Tables

**Table 1:** Isoelectric points of amino acids [52] and SMH in solutions of these amino acids.

Amino Acid	pI of Amino Acid	pI of SMH
Glycine	6	3.65 ± 0.3 ^1^
Valin	6	3.8 ± 0.5
Phenylalanine	5.5	4 ± 0.5
Serin	5.7	3.35 ± 0.2
Glutamic acid	3.2	3.45 ± 0.4
Arginine (hydrochloride)	10.8	4.0 ± 0.5
Methionine	5.75	2.4 ± 0.5

^1^ The spread corresponds to a 95% confidence interval.

**Table 2:** ζ-potential (mV) of SMH in solutions of glucose, sucrose, ATP and seven amino acids at pH = 7 in water and with the addition of 150 mM KCl.

Biomolecules in Solution	pH = 7, Water	pH = 7, 150 mM KCl Solution
Glycine	−22.3 ± 4.3	−17.3 ± 4.8 ^1^
Valin	−31.1 ± 4.3	−25.0 ± 3.8
Phenylalanine	−16.6 ± 3.3	−9.5 ± 3.1
Serin	−29.7 ± 4.4	−24.4 ± 2.7
Glutamic acid	−21.3 ± 4.9	−17.4 ± 2.6
Arginine (hydrochloride)	−13.7 ± 3.9	−9.7 ± 2.9
Methionine	−28.8 ± 3.5	−24.6 ± 2.8
ATP	−22.1 ± 2.7	−20.3 ± 3.2
Glucose	−20.5 ± 3.9	−14.9 ± 3.9
Sucrose	−19.6 ± 4.5	−11.4 ± 3.6

^1^ The spread corresponds to a 95% confidence interval.

## Data Availability

Data is contained within the article or Supplementary Material.

## Supplementary Materials

The following supporting information can be downloaded at https://www.mdpi.com/article/10.3390/ijms27136015/s1.

## Abbreviations

The following abbreviations are used in this manuscript:

SMH	Submicron heterogeneities
